# iPS cell technologies: significance and applications to CNS regeneration and disease

**DOI:** 10.1186/1756-6606-7-22

**Published:** 2014-03-31

**Authors:** Hideyuki Okano, Shinya Yamanaka

**Affiliations:** 1 Department of Physiology, Keio University School of Medicine, 35 Shinanomachi, Shinjuku-ku, Tokyo 160-8582, Japan; 2 Center for iPS Cell Research & Application, Kyoto University, 53 Kawaharacho, Shogoin, Sakyoku, Kyoto 606-8507, Japan; 3 Gladstone Institute of Cardiovascular Disease, San Francisco, CA 94158, USA

**Keywords:** Induced pluripotent stem cell, Cell transplantation, Spinal cord injury, Modeling human diseases, Parkinson’s disease, Alzheimer’s disease

## Abstract

In 2006, we demonstrated that mature somatic cells can be reprogrammed to a pluripotent state by gene transfer, generating induced pluripotent stem (iPS) cells. Since that time, there has been an enormous increase in interest regarding the application of iPS cell technologies to medical science, in particular for regenerative medicine and human disease modeling. In this review article, we outline the current status of applications of iPS technology to cell therapies (particularly for spinal cord injury), as well as neurological disease-specific iPS cell research (particularly for Parkinson’s disease and Alzheimer’s disease). Finally, future directions of iPS cell research are discussed including a) development of an accurate assay system for disease-associated phenotypes, b) demonstration of causative relationships between genotypes and phenotypes by genome editing, c) application to sporadic and common diseases, and d) application to preemptive medicine.

## The development of induced pluripotent stem (iPS) cell technologies and their significance

The 2012 Nobel Prize in Physiology or Medicine was awarded for “The discovery that mature cells can be reprogrammed to become pluripotent.” First, we would like to consider the significance of this research. The lives of mammals, including humans, begin with the fertilization of an egg by a sperm cell. In humans, a blastocyst composed of 70-100 cells forms by approximately 5.5 days after fertilization. The blastocyst is composed of the inner cell mass, the cell population that has the ability to differentiate into the various cells that constitute the body (pluripotency), and the trophoblast, the cells that develop into the placenta and extra-embryonic tissues and do not contribute cells to the body. In the embryonic stage, the pluripotent cells of the inner cell mass differentiate into the three germ layers, endoderm, mesoderm, and ectoderm, which will form specific organs and tissues containing somatic stem cells with limited differentiation potencies. These somatic stem cells continue to divide and differentiate, and, by adulthood, an individual composed of 60 trillion cells is produced. Somatic stem cells born in the fetal period actively divide, and are involved in the formation and growth of various organs. However, even in the adult, somatic stem cells persist in niches in every organ and tissue, and play an important role in maintaining organ and tissue homeostasis. When cells in the inner cell mass are removed at the blastocyst stage and cultured *in vitro*, pluripotent embryonic stem (ES) cells are obtained. Thus, in the normal process of development, cell differentiation of the three germ layers proceeds from the simple stages of the fertilized egg and blastocyst, and ultimately produces an individual consisting of a complex cellular society.

In 1893, August Weismann argued that only germ cells (eggs and spermatozoa) maintain a “determinant,” which was described as heritable information essential to decide on the functions and features of all somatic cells in the body [1]. In his germ plasm theory, the determinants are lost or irreversibly inactivated in differentiated somatic cells.

It took more than 50 years for researchers to rewrite this dogma. In 1962, Sir John Gurdon demonstrated the acquisition of pluripotency by reprogramming cells to their initial stage using a novel research technique, i.e., producing cloned individuals by transferring somatic cell nuclei into eggs [2]. However, for many years, that result was regarded as a special case limited to frogs alone. The production of Dolly the sheep by transferring the nucleus of a somatic cell (mammary gland epithelial cell) by Sir Ian Wilmut in the late 1990s [3] showed that cloning could also be applied to mammals.

These brilliant previous works led to our studies that culminated in the induction of pluripotency in mouse somatic cells in 2006, using retroviral vectors to introduce four genes that encode transcription factors i.e., *Oct4*, *Sox2*, *Klf4*, and *c-Myc*. We designated these cells as iPS cells [4]. In 2007, we succeeded in generating human iPS cells using genes encoding the same four transcription factors [1]. The results of this research showed that although the developmental process was thought to be irreversible, by introducing key genes into differentiated adult cells the cells could be reset to a state in the extremely early stage of development in which they possessed pluripotency. That is, the results demonstrated that the differentiation process was reversible. This startling discovery made it necessary to rewrite the embryology textbooks.

**Figure 1:**
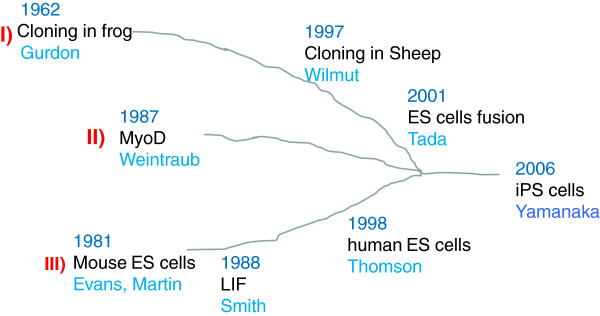
**The history of investigations of cellular reprogramming that led to the development of iPS cells.** Our generation of iPS cells in 2006 [4] became possible due to three scientific lines of investigation: 1) nuclear reprogramming, 2) factor-mediated cell fate conversion, and 3) ES cells. See the text for details (modified from Reference [5] with permission).

Three major lines of research led us to the production of iPS cells [5] (Figure 1). The first, as described above, was nuclear reprogramming initiated by Sir John Gurdon in his research of cloning frogs by nuclear transfer in 1962 [2] and by Sir Ian Wilmut, who cloned a mammal for the first time in 1997 [3]. In addition, Takashi Tada showed that mouse ES cells contain factors that induce reprogramming in 2001 [6]. The second line of research was factor-mediated cell fate conversion, initiated by Harold Weintraub, who showed that fibroblasts can be converted into the muscle lineage by transduction with the *MyoD* gene, which encodes a muscle lineage-specific basic helix-loop-helix transcription factor in 1987 [7]. The third line of research was the development of mouse ES cells, initiated by Sir Martin Evans and Gail Martin in 1981 [8,9]. Austin Smith established culture conditions for mouse ES cells and identified many factors essential for pluripotency including leukemia inhibitory factor (LIF) in 1988 [10]. Later, he developed the method to induce the ground state of mouse ES cell self-renewal using inhibitors for mitogen-activated protein kinase and glycogen synthase kinase 3 [11], which supports the establishment of fully reprogrammed mouse iPS cells. Furthermore, James Thomson generated human ES cells [12] and established their optimal culture conditions using fibroblast growth factor-2 (FGF-2). Without these previous studies, we could never have generated iPS cells. Interest rapidly escalated, and, in tandem with the birth of iPS cell technology, pluripotency leapt into the mainstream of life sciences research in the form of “reprogramming technology” [13]. However, there remain many unanswered questions regarding reprogramming technology. What are the reprogramming factors in the egg cytoplasm that are active in cloning technology? What do they have in common with the factors required to establish iPS cells and what are the differences? What kind of epigenetic changes occur in association with the reprogramming?

**Figure 2:**
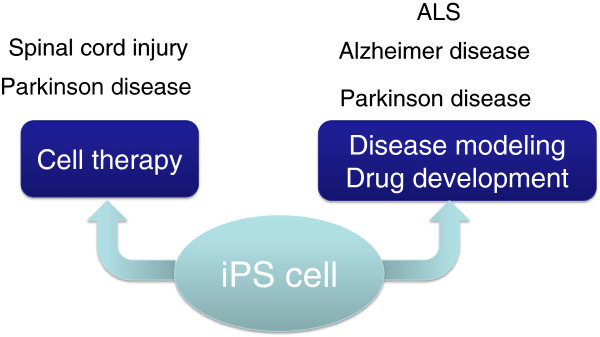
**The application of iPS cell technologies to medical science.** iPS cell technologies can be used for medical science including 1) cell therapies and 2) disease modeling or drug development. See the text for details.

Apart from basic research in embryology, broad interest has been drawn to the following possible applications of iPS cell research: (1) regenerative medicine, including elucidating disease pathologies and drug discovery research using iPS cell disease models, and (2) medical treatments (Figure 2). In this review, we describe these potential applications in the context of the results of our own research.

## Applications of iPS cell technologies to regenerative medicine

### General statement of iPS-based cell therapy

iPS cells can be prepared from patients themselves and therefore great expectations have been placed on iPS cell technology because regenerative medicine can be implemented in the form of autografts presumably without any graft rejection reactions. Although there have been some controversies [14], the immunogenicity of terminally differentiated cells derived from iPS cells can be negligible [15-17]. Moreover, there has been substantial interest in the possibility of regenerative medicine without using the patient’s own cells; that is, using iPS cell stocks that have been established from donor somatic cells that are homozygous at the three major human leukocyte antigen (HLA) gene loci and match the patient’s HLA type [18]. The development of regenerative medicine using iPS cells is being pursued in Japan and the USA for the treatment of patients with retinal diseases, including age-related macular degeneration [19], spinal cord injuries [17], Parkinson’s disease (PD) [20,21], corneal diseases [22-24], myocardial infarction [25,26], diseases that cause thrombocytopenia, including aplastic anemia and leukemia [27,28], as well as diseases such as multiple sclerosis (MS) and recessive dystrophic epidermolysis bullosa [29] (Table 1).

**Table 1:** Planned clinical trials of iPS cell-based therapies

***Principal investigator (Institute/Location)***	***Cell type to transplant***	***Target disorders***
Masayo Takahashi, (RIKEN)	Retinal Pigment Epithelium (sheet)	Age-related macular degeneration (wet type)
Alfred Lane, Anthony Oro, Marius Wernig (Stanford University)	Keratinocytes	Recessive dystrophic epidermolysis bullosa (RDEB)
Mahendra Rao (NIH)	DA neurons	Parkinson’s disease
Koji Eto (Kyoto University)	Megakaryocyte	Thrombocytopenia
Jun Takahashi (Kyoto University)	DA neurons	Parkinson’s disease
Steve Goldman, (University of Rochester)	Oligodendrocyte precursor cell	Multiple Sclerosis
Hideyuki Okano, Masaya Nakamura (Keio University)	Neural stem/progenitor cells	Spinal Cord Injury
Shigeto Shimmura (Keio University)	Corneal endothelial cells	Corneal endothelial dysfunction
Koji Nishida (Osaka University)	Corneal epithelial cells (sheet)	Corneal epithelial dysfunction and trauma (e.g. Stevens–Johnson syndrome)
Yoshiki Sawa (Osaka University)	Cardiomyocytes (sheet)	Heart Failure
Keiichi Fukuda (Keio University)	Cardiomyocytes (sphere)	Heart Failure
Yoshiki Sasai and Masayo Takahashi (RIKEN)	Neuroretinal sheet including photoreceptor cells	Retinitis pigmentosa
Advanced Cell Technology	Megakaryocytes	Refractory thrombocytopenia

Representative studies of iPS-based cell therapy with planned clinical trials are listed.

References: [17,19-29].

### Regenerative medicine research to discover a treatment for spinal cord injury (SCI) by means of iPS cell technologies

In 1998, Hideyuki Okano, in collaboration with Steven Goldman, demonstrated for the first time the presence of neural stem/progenitor cells (NS/PCs) in the adult human brain using a neural stem cell marker, the ribonucleic acid (RNA)-binding protein Musashi1 [30,31]. Research on nerve regeneration then commenced in earnest. That same year, we began regenerative medicine research on neural stem cell transplantation in a rat model of SCI, and have since made progress in developing NS/PC transplantation therapies in experiments on animal models of SCI. First, motor function was restored by transplanting rat fetal central nervous system (CNS)-derived NS/PCs into a rat SCI model [32]. The same study also showed that the sub-acute phase is the optimal time for NS/PC transplantation after SCI. In this study, at least part of the putative mechanism by which NS/PC transplantation restored function was identified in animal models of SCI. Both the cell autonomous effect (such as synaptogenesis between graft-derived neurons and host-derived neurons) and non-cell autonomous (trophic) effects mediated cytokines released from the graft-derived cells are likely contributing to tissue repair and functional recovery. Subsequently, a non-human primate SCI model was developed using the common marmoset, and motor function in that model was restored by transplanting human fetal CNS-derived stem cells [33]. In the same study, a behavioral assay for motor function associated with SCI was developed. Based on these studies, a preclinical research system for cell transplantation therapy was established in a non-human primate SCI model.

Given these findings, we began preparations for clinical studies of human fetal CNS-derived NS/PC transplantation to treat SCI patients. However, with the guidelines for clinical research on human stem cells of the Japanese Ministry of Health, Labor and Welfare that came into effect in 2006, human fetus-derived cells and ES cells became ineligible for use in regenerative medicine. Thus, we had no choice but to change our strategy (human ES cells became eligible for use in the 2013 guidelines). In 2006, one of our research groups (Yamanaka’s group) established iPS cells from adult mouse skin cells. Hypothesizing that it might be possible to induce NS/PCs from iPS cells, we (Okano’s group) turned our attention to iPS cells as a means of obtaining NS/PCs without using fetal or ES cells. Based on conditions that were developed for experiments on mouse ES cells [34,35], NS/PCs were induced from mouse iPS cells [36]. The following year, we succeeded in restoring motor function by transplanting these mouse iPS cell-derived NS/PCs into a mouse model of SCI, and reported that when “good iPS cells” -derived NS/PCs, which had been pre-evaluated as non-tumorigenic by the transplantation into the brains of immunocompromised mice, were used for transplantation, motor function was restored for a long period of time without tumors developing [37]. In 2011, we succeeded in restoring motor function by transplanting human iPS cell-derived stem cells into a mouse SCI model [38]. Moreover, in 2012, motor function was restored by transplanting human iPS (line 201B7) cell-derived NS/PCs into the marmoset SCI model, and long-term motor function was recovered without observable tumor formation [39]. This finding was of great significance in terms of preclinical research, and provided a proof of concept that could potentially lead to a treatment method.

Collectively, when mouse or human iPS cells were induced to form NS/PCs and were transplanted into mouse or non-human primate SCI models, long-term restoration of motor function was induced, without tumorigenicity, by selecting a suitable iPS cell line [17,40]. Considering the sub-acute phase (2-4 weeks after the injury) as the optimal time for iPS cells-derived NS/PCs transplantation for SCI patients, there are following major difficulties with autograft-based cell therapy. First, it takes about a few months to establish iPS cells. Second, it also takes three months to induce them into NS/PCs *in vitro*. Third, one more year would be required for the quality control including their tumorigenesis.

**Figure 3:**
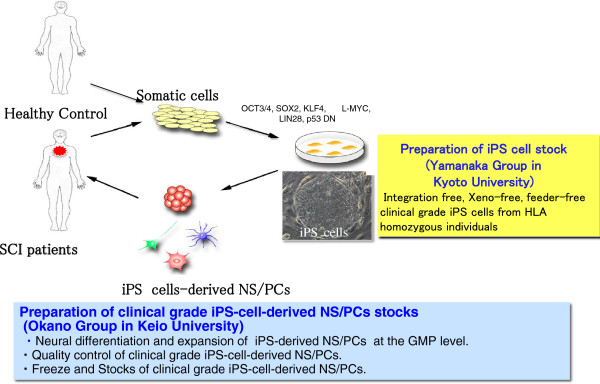
**Strategies for the development of iPS cell-based cell therapy for SCI patients.** Our collaborative team (Okano’s group at Keio University and Yamanaka’s group at Kyoto University) have been developing an iPS cell-based cell therapy for SCI since 2006. Our previous preclinical studies have shown that long-term functional restoration can be obtained by transplantation of NS/PCs derived from appropriate iPS cells clones without observable tumor formation [10]. Currently, we aim to develop iPS cells-based cell therapy for SCI patients at sub-acute phase using the clinical grade iPS cell-derived NS/PCs (i.e., the role of Okano’s group described in the blue box) which have been prepared from human iPS cell stock (i.e., the role of Yamanaka’s group described in the yellow box).

Considering these, our collaborative team (Okano and Yamanaka laboratories) are currently planning iPS-based cell therapy for SCI patients in the sub-acute phase using clinical-grade integration-free human iPS cell lines that will be generated by Kyoto University’s Center for iPS Cell Research and Application (CiRA). We will establish a production method, as well as a storage and management system, for human iPS cell-derived NS/PCs for use in clinical research for spinal cord regeneration, build an iPS cell-derived NS/PC stock for regenerative medicine, establish safety screenings against post-transplantation neoplastic transformation, and commence clinical research (Phase I–IIa) trials for the treatment of sub-acute phase SCI (Figure 3). As these studies progress, the application of iPS cells to treat chronic phase SCI and stroke will be investigated. Significant therapeutic efficacy in the treatment of chronic phase SCI has not been achieved by cell transplantation alone [41]. However, clinical studies are planned using antagonists of axon growth inhibitors, such as Semaphorin3A inhibitors [42], followed by multidisciplinary rehabilitation combination therapies. We aim to perform a clinical trial based on the Pharmaceutical Affairs Act in collaboration with drug companies and to use iPS cell-derived NS/PC stocks for regenerative medicine to establish treatment methods for stroke, MS, and Huntington’s disease.

## iPS cell technologies in nervous system disease research

### General statement of human disease modeling with iPS cell technologies

Lesion sites are difficult to access in patients with degenerative diseases of the nervous system. Therefore, in past studies, cell biological or biochemical analyses of their pathology centered on forced expression of the causative genes in non-nervous system cultured cell lines and on mice in which the causative gene was knocked out. However, in a few instances, the animal or cell models did not necessarily reflect the human pathology. Identifying cell biological or biochemical changes in the initial stages of the disease, before onset of symptoms, has been difficult given analyses conducted on postmortem brains. However, with the development of iPS cell technologies, it became possible to establish pluripotent stem cells from the somatic cells of anyone, irrespective of race, genetic background, or whether the person exhibits disease symptoms. Thus, it is no exaggeration to say that generation of disease-specific iPS cells using iPS cell technologies is the sole means of reproducing *ex vivo* phenomena that occur in patients *in vivo*, particularly for nervous system disorders. The result has been a tremendous desire by investigators who are conducting research on neurological diseases to become engaged in disease-specific iPS cell research [43-45].

A variety of disease-specific iPS cells have been used to study nervous system diseases, including amyotrophic lateral sclerosis (ALS) [46-48], spinal muscular atrophy [49], spinobulbar muscular atrophy [50], Friedreich’s ataxia [51], Alzheimer’s disease (AD) [52-54], PD [55-58], Huntington’s disease [59,60], Machado-Joseph disease [61], fragile X-syndrome [62], Rett’s syndrome [63], familial dysautonomia (FD) [64], Pelizaeus-Merzbacher disease [65], adrenoleukodystrophy [66], schizophrenia [67-69], and Dravet’s syndrome of intractable epilepsy [70-72] (Table 2). In the following sections, we describe the results of nervous system disease-specific iPS cell research using PD and AD as examples [44].

**Table 2:** Representative reports on neurological/psychiatric disorders

***Name of disease***	***Gene responsible***	***Cells responsible for pathogenesis***	***References***
		**Neurodevelopmental disorders**	
*Rett syndrome*	MeCP2, CDK5L5	Neurons, neural precursors	[63]
*Spinomuscular atrophy*	SMN1	Motor neurons	[49]
*Familial dysautonomia*	IKBKAP	Neural crest precursor cells	[64]
*Fragile X-syndrome*	FMR1	Neurons	[62]
*Adrenoleuko- dystrophy*	ABCD1	Oligodendrocytes	[66]
*Pelizaeus- Merzbacher disease*	PLP1	Oligodendrocytes	[65]
*Dravet syndrome*	SCN1A	Neurons	[70-72]
		**Late-onset neurodegenerative disorders**	
*Alzheimer’s disease*	PS1, PS2, APP, sporadic	Neurons	[52,53,58,100]
*Parkinson’s disease*	α-synuclein, PARKIN, PINK-1, LRRK2 etc. sporadic	Dopaminergic neurons	[55-59,83,87]
*Amyotrophic lateral sclerosis*	SOD1, TDP43, FUS, C9ORF etc. sporadic	Motor neurons, astroglia	[47-49]
*Spinobulbar muscular atrophy*	Androgen receptor	Motor neurons, skeletal muscles	[50]
*Huntington’s disease*	HTT	Glutamatergic neurons, GABAergic neurons	[59,60]
*Machado-Joseph disease*	ATX3	Glutamatergic neurons	[61]
		**Psychiatric disorders**	
*Schizophrenia*	22q11.2, sporadic	Glutamatergic neurons, GABAergic neurons, dopaminergic neurons, etc.	[67-69]

Extracted and modified from References [43,44] and [73].

### Modeling PD with disease-specific iPS cells

PD is the second most common neurodegenerative disease after AD. More than 4 million patients are afflicted with PD globally. In Japan, its prevalence is about 100–150 cases per 0.5 million population [73]. PD is characterized by selective degeneration of dopaminergic (DA) neurons (A9 neurons) in the substantia nigra, which results in motor symptoms, including tremor, rigidity, akinesia, and postural instability. The remarkable loss of neuromelanin-containing DA neurons in the substantia nigra pars compacta and the appearance of Lewy bodies (i.e., eosinophilic intracellular proteinaceous inclusions) are characteristic of PD and related diseases [74].

L-Dopa therapy or deep brain stimulation are current methods of treating PD, but they are not curative treatments. In most patients, the onset of PD occurs when the person is in their late 60s to early 70s; therefore, treatment of PD has become a major task in countries facing an aging society. Based on previous research, mutation of a specific gene is the cause of PD (familial PD [FPD]) in approximately 10% of PD patients, whereas the other 90% of patients have sporadic PD [73].

Interplay between genetic and environmental factors is likely to play an important role in the pathogenesis of PD. In human molecular genetic studies of rare monogenic forms of PD (FPD), at least 18 loci and 11 genes leading to the development of FPD have been identified. FPD-associated loci and genes for which there is conclusive evidence of a role in the disease mechanism include PARK1/PARK4 (α-synuclein [SNCA]), PARK2 (Parkin), PARK6 (PTEN-induced kinase 1 [PINK-1]), PRAK7 (DJ-1), PARK8 (leucine-rich repeat kinase 2 [LRRK2]), and PARK9 (ATPase type 13A [ATP13A2]). The gene products are closely associated with the regulation of mitochondrial function and oxidative stress. Environmental risk factors for PD include drinking well-water, and exposure to pesticides, herbicides, and heavy metals (Fe, Cu, and Zn). Furthermore, parkinsonism can be experimentally induced by administration of certain drugs, including MPTP (1-methyl-4-phenyl-1,2,3,6-tetrahydropyridine), which is metabolized to MPP^+^ in glial cells and blocks mitochondrial functions. The mechanisms common to the involvement of both genetic factors and environmental factors in causing PD are mitochondrial dysfunctions and increased oxidative stress [73]. Notably, there are close associations between mitochondrial homeostatic mechanisms and the gene products encoded by the genetic loci that are correlated with FPD. DA neurons are constitutively exposed to oxidative stress, which damages mitochondria and impairs membrane depolarization. The lowered membrane potential of mitochondria leads to stabilization of the protein kinase PINK1 (PARK6) [75]. The stabilized PINK-1 then phosphorylates the PARKIN protein (PARK2) (a member of the E3 ubiquitin ligase family) [76-78] and recruits it from the cytoplasm to the outer membrane of the mitochondria, where it ubiquitinates mitochondrial outer membrane proteins, including VDAC1 [79]. Mitochondrial outer membrane proteins thus tagged are recognized by isolation membranes, which then fuse with lysosomes and are ultimately degraded by lysosomal enzymes. In addition, ATP13A2 (PARK9) [80] is an H^+^-ATPase involved in lysosomal acidification, which is necessary for lysosome function. Mitochondria damaged by exposure to oxidative stress are degraded by mitophagy. The mutations in FPD result in impaired mitochondrial homeostatic mechanisms. We have generated iPS cells from FPD patients [58] to clarify the interaction between impaired mitochondrial homeostatic mechanisms and the development of PD. Okano’s group has established iPS cells from cutaneous fibroblasts obtained from patients with the PARK2 form of FPD (Patient A: female with an exon 2–4 deletion mutation; Patient B: male with an exon 6–7 deletion mutation) by performing retroviral gene transduction (*Oct4*, *Sox2, Klf4*, and *c-Myc*). After selecting seven clones with good neuronal differentiation (PA1, PA9, PA22, PB1, PB2, PB17, and PB20), NS/PCs and tyrosine hydroxylase (TH)-positive DA neurons were induced from PARK2 patient-derived iPS cells and, as a control, from human ES cells and healthy adult-derived iPS cells. In investigating PARK2, patient-derived fibroblasts, iPS cells, NS/PCs, neurons, and DA neurons were used to examine multiple aspects, including the transcriptome, metabolome, proteome, and mitochondrial homeostasis [58,73].

Increased oxidative stress is usually involved in the pathogenesis of PD; therefore, oxidative stress was measured in PARK2 iPS cell-derived neurons. These cells exhibited increased oxidative stress accompanied by activation of the Nrf2 pathway, which exerts a cytoprotective role under conditions of reactive oxygen species accumulation. A metabolome analysis of glycolytic pathways, the tricarboxylic acid cycle, and pentose phosphate pathways suggested that mitochondrial were dysfunctional in the PARK2-derived neurons (H.O., unpublished results). Further characterization of mitochondria in the PARK2 iPS cell-derived neurons revealed abnormal mitochondrial morphology and impaired mitochondrial turnover.

Lewy bodies, a pathology characteristic of PD, and their main component, α-synuclein, were investigated in PARK2 patient-derived iPS cells [58]. Based on an analysis of patient brain autopsies, Lewy bodies were found to accumulate in the neurons of patients with sporadic PD. However, while there have been several autopsy reports for brains of PARK2 patients, α-synuclein was not generally thought to accumulate in the brain in PARK2 FPD. When the postmortem brain of a PARK2 patient was examined histologically, Lewy bodies and aggregates of α-synuclein were confirmed, and examination of neuronal cells derived from iPS cells of the same patient revealed that α-synuclein had accumulated in a similar manner [58]. These results were the first to demonstrate that patient iPS cell-derived neuronal cells faithfully reproduced a phenomenon that occurred in the brain of the same patient.

There is growing interest in genome editing of human iPS cells in introducing mutations into isogenic iPS cells (reviewed in [81]) and in rescue experiments via genetic repair, as methods to demonstrate genotype-phenotype causal relationships in human genetic disorders (discussed in iPS cell technologies in nervous system disease research and Conclusion) (Figure 2). Various PD-associated abnormalities, including neurite outgrowth abnormalities, DA neuron death induced by the addition of 6-hydroxydopamine, tau and α-synuclein deposition, and gene expression changes in DA neurons, have been observed in DA neurons derived from iPS cells prepared from a patient with a LRRK2 gene mutation (G2019S mutation) and in isogenic control iPS cells in which the G2019S mutation was introduced. These PD-associated phenotypes were rescued by genetic correction of the LRRK2 mutation in the patient-derived iPS cells [82].

Studies of PD using iPS cell technology have shown the presence of PD-associated abnormalities in: 1) mitochondrial function, 2) the unfolded protein response and Golgi to endoplasmic reticulum transport and 3) axonal transport and cytoskeletal and neurite extension/retraction responses (Courtesy of Dr. Ole Isacson, Harvard Medical School). Thus, iPS-cell-based disease modeling is expected to contribute to the elucidation of the pathophysiology of PD, and be useful in drug screening and the development of methods for extremely early diagnosis, before the appearance of motor symptoms.

### AD

AD accounts for approximately half of all cases of dementia and is the most common intractable neurological disease. AD is usually first manifested by a memory disorder in a person aged 65 years or older. AD always progresses to disorientation and a decreased ability to comprehend and make judgments, and ultimately leads to personality disorders and a bedfast state. In recent years, early-onset AD, in which onset occurs from the fifth decade of life onward, has also attracted attention. People with early-onset AD lose the ability to conduct their daily lives and need long-term care; therefore, this disease has become a major social problem. Current treatment is primarily symptomatic, and the development of curative treatments has been slow. There are currently no prospects for a complete cure [44].

Based on previous research, it is clear that large amounts of amyloid beta (Aβ) accumulate in the brains of AD patients and cause pathological changes called senile plaques. Moreover, experiments conducted on cell cultures and mice suggest that the highly toxic Aβ-42 may be overproduced in AD. The “amyloid hypothesis”, which states that Aβ is the cause of the disease, has been difficult to verify in living nerve cells of patients using previous technologies. In collaboration with the Keio University neurology department team (Drs. Takuya Yagi and Daisuke Ito, Professor Norihiro Suzuki, and colleagues), we produced iPS cells from skin fibroblasts of familial AD (FAD) patients (presenilin (PS)-1 or -2 mutations) and succeeded in inducing neuronal cells for the first time [52]. We confirmed that these patient-derived neuronal cells produce twice the normal level of the highly toxic Aβ-42. This result correlated with Aβ accumulation in neural cells derived from living patients with AD. In addition, we treated AD iPS cell-derived neuronal cells with a γ-secretase modulator, which is a candidate drug for the treatment of AD, and showed that production of Aβ-42 was inhibited. Thus, these disease-specific iPS cells enabled a novel drug to be developed for the treatment of dementia [44].

In a study by Israel et al., Aβ accumulation in neural cells induced from sporadic AD patient-derived iPS cells [53] was similar to our finding showing that in neural cells induced from FAD patient-derived iPS cells [52]. Accumulation of phosphorylated tau, in addition to Aβ, was observed in the neurons induced from iPS cells of one of the sporadic AD patients [53]. Gene analysis data were not described in this report [53], but the phosphorylated tau phenotype was not detected in FAD-derived neurons that had a PS1 or PS2 gene mutation [52]. These observations are extremely interesting from the standpoint of the diversity of AD phenotypes. Based on these results, AD pathology can be detected in sporadic AD, as well as FAD, and will lead to the development of new treatments [44].

In addition, Dr. Haruhisa Inoue’s research group at CiRA produced iPS cells from the skin cells of patients with a mutation in the amyloid precursor protein (APP) gene, which is a causative gene in early-onset (familial) AD, and from patients with late-onset (sporadic) AD who had no family history of AD. The mutant APP iPS cells were induced to differentiate into cerebral neurons. When a mutation called APP-E693Δ was present, the Aβ protein formed oligomers, accumulated in cells, induced endoplasmic reticulum and oxidative stress, and caused induction of the cell death gene Caspase4. Intracellular stress and neuronal cell death were inhibited by the unsaturated fatty acid docosahexaenoic acid (DHA), which is present at high levels in fish oils. Moreover, intracellular Aβ oligomers and cell stress have also been observed in some patients with late-onset sporadic AD, similar to the APP-E693Δ cells. Based on the analysis of neurons induced from iPS cells derived from several patients, there is an AD population in which DHA is effective and an AD population in which is it not. These findings suggest a diversity of pathologies in AD and, correspondingly, the need for a diversity of treatment strategies [44,54].

Monogenic FAD is rare in comparison to the sporadic form. However, genetic predisposing factors, for example ApoE, are present in sporadic AD. ApoE4 is the major known genetic risk factor for AD. ApoE is one of the five main types of blood lipoproteins (A-E) with 299 amino acids, with three different isoforms (ApoE2 (Cys112, Cys158), ApoE3 (Cys112, Arg158) and ApoE4 (Arg112, Arg158)) [83]. Genetic polymorphism of the *ApoE* gene is responsible for the generation of these isoforms [84]. While *ApoE4* allele is found in approximately 14% of the population [85], the *ApoE4* allele is genetically associated with late-onset familial and sporadic forms of AD [86], highlighting the importance of *ApoE4* in the pathogenesis of AD. In the future, the role of *ApoE4* should be characterized by a combination of iPS cell technologies and genome editing.

### Future tasks for iPS cell researchers with regard to modeling human diseases

As described above, iPS cell researchers have developed new strategies to study the pathophysiology of human diseases and to provide assay systems for drug screening. However, many tasks remain to be accomplished to enable iPS cell technologies to accurately model human diseases and to develop new therapeutic interventions.

a) **Development of an accurate assay system for disease-associated phenotypes**

One of the problems with current iPS cell technologies is that the somatic cells generated from undifferentiated iPS cells remain immature for long periods. As a result, iPS cell technologies have been most successful in modeling pediatric or early-onset diseases, including FD [64], epilepsy (e.g., Dravet syndrome [70-72]), and Rett syndrome [63]. A potential problem with current iPS cell technologies in modeling late-onset neurodegenerative diseases is the difficulty in obtaining age-related phenotypes in a relatively short timeframe. Lorenz Studer’s group recently succeeded in inducing rapid cell aging by mis-expression of progerin, a truncated form of lamin A that is associated with premature aging [87]. This method made it possible to induce aging in PD patient iPS cell-derived DA neurons that resulted in disease-related phenotypes, including severe dendrite degeneration, progressive loss of TH-positive cells, and abnormal mitochondria or Lewy body precursor-like inclusions, which are difficult to identify using conventional neuronal differentiation methods for iPS cells [87]. Notably, we did not observe any Lewy bodies in DA neurons induced from PD patient-derived iPS cells [58], even though α-synuclein accumulated in progerin-expressing DA neurons *in vitro*[87] and Lewy bodies were prominent in the brain autopsy of the patient [58]. Although α-synuclein is their major component, Lewy bodies contain several other components including neurofilaments and ubiquitin [74,88,89]. These observations indicate that Lewy body formation is a dystrophic age-related event, and, thus, Lewy bodies may not form in DA neurons induced from PD patient-derived iPS cells by conventional culture methods. Thus, progerin-induced aging is a versatile method to investigate the features of late-onset age-related diseases by iPS cell-based disease modeling, while their application to other age-related diseases needs to be verified.

b) **Demonstration of causative relationships between genotypes and phenotypes by genome editing**

As a result of the rapid progress in human genome sequencing since the advent of next-generation sequencers, enormous numbers of disease-related mutations and single-nucleotide polymorphisms (SNPs) have been identified. Heterogeneity in the genetic mutations associated with some diseases is common. In most diseases, there is no formal proof of a causal relationship between the genetic mutation and the disease phenotype because experimental genetic studies are not possible in humans, as opposed to animal models, such as *Drosophila* and mouse. However, demonstration of a causal relationship between a genetic mutation and a disease phenotype can be verified using genome editing technologies such as the helper-dependent adenoviral vector [90], zinc-finger nucleases [91], transcription activator-like effector nucleases [92,93], or the CRISPR-Cas9 system [94,95] or its improved method [96]. These technologies can be used to perform rescue experiments with gene corrections, as well as recapitulation of the disease phenotype by introducing disease-related mutations into control iPS cells [82]. Thus, while genome editing is a powerful technology for demonstrating genotype-phenotype causal relationships, the current genome editing techniques can only be applied to monogenic disorders, and new technologies will need to be developed to investigate polygenic disorders.

c) **Application to sporadic and common diseases**

Although Mendelian inheritance patterns have been well documented in several neurodegenerative diseases, including PD, AD, and ALS, the majority of the cases of these diseases are sporadic, and the genetic defects responsible for these cases remain to be identified [97]. In Mendelian diseases, the effect of genetic variation is extremely large, while the allele frequency is extremely low. Thus, Mendelian diseases are well suited for iPS cell-based disease modeling and genome editing. By contrast, the molecular etiology of most sporadic neurodegenerative diseases remains unknown. In a series of studies of the human genetics of sporadic diseases, genome-wide association studies (GWAS) with SNPs have been conducted as a means of identifying susceptibility genes for sporadic neurodegenerative diseases. Remarkably, these GWAS studies have succeeded in identifying disease-related rare variants with a high odds ratio [97]. For example, the glucocerebrosidase (GBA) gene polymorphism was identified as a robust genetic risk factor for PD [98]. It will be important to characterize the role of GBA mutations from the standpoint of the molecular etiology of PD, using iPS cell-based *in vitro* characterization. Since their effect size is not small, such sporadic diseases with rare genetic variants are also likely to be suitable targets for iPS cell-based disease modeling. However, it will be important to establish iPS cells from a sufficient number of patients and to characterize a large number of clones to perform statistical analyses. Therefore, it will be essential to develop large-scale automated systems for the production and differentiation of iPS cells.

d) **Application to preemptive medicine**

iPS cell-based disease modeling could play an important role in the early diagnosis of late-onset neurodegenerative diseases such as AD and PD. Since the motor symptoms of PD do not develop until almost 70% of the DA neurons in the substantia nigra have been lost, the molecular mechanisms that predominate during the initial stages of PD remain unknown. However, studies characterizing iPS cells derived from the somatic cells of PD patients have provided an excellent opportunity and excellent tools to investigate the course of changes during PD, from the asymptomatic phases through to the later stages when the pathology has become prominent. Such studies could help to develop an appropriate preemptive neuroprotective treatment for PD, including small molecules, gene therapy, or cell therapy, which could be started early in the asymptomatic phase. AD usually has a long progression of more than 30 years that consists of an asymptomatic phase of ~20 years, a mild cognitive impairment (MCI) phase of ~10 years, and a dementia phase of unlimited length. Amyloid plaques form and continue to enlarge in the asymptomatic phase, and there is already substantial neuronal loss and brain atrophy in the MCI phase [99]. Thus, if diagnosis were possible in the asymptomatic phase, it would provide a great advantage by enabling the use of treatments to prevent dementia, including γ-secretase modulators [52], Non-Steroidal Anti-Inflammatory Drugs (NSAIDs) [100], and DHA [54]. A combination of iPS cell-based phenotypic screening, whole genome sequencing by next-generation sequencing to identify AD-related polymorphisms, and imaging of Aβ and tau by positron emission tomography would enable reliable diagnosis of AD in the asymptomatic phase. While obtaining a proof of concept for such preemptive treatments of AD would be difficult to obtain in a short time, we hope that such data can be obtained by using a combination of large-scale iPS cell-based disease modeling and a cohort study of dominantly inherited FAD, similar to the Dominantly Inherited Alzheimer Network (DIAN) study [101-103]. The development of preemptive treatments for late-onset neurodegenerative diseases would be enormously important in rapidly aging countries like Japan.

## Conclusions

As described above, since 2006, there have been enormous progresses in iPS cell technologies aiming for medical science, in both regenerative medicine and human disease modeling. Furthermore, iPS cell technologies could be applied for preemptive medicine. However, it is also true that iPS cell technologies have not yet saved any patients’ lives at this moment in early 2014. Continuous efforts through the cooperation of basic stem cell biology, clinical investigation of diseases, translational research, pharmaceutical science, regulatory science and system biology will be necessary to let iPS cells really contribute to human health.

## Competing interests

H.O. is a paid scientific consultant to San Bio, Inc., Eisai Co., Ltd., and Daiichi Sankyo Co., Ltd. S.Y. is a member without salary of the scientific advisory boards of iPierian, iPS Academia Japan, Megakaryon Corporation and HEALIOS K.K. Japan.

## Authors’ contributions

Both HO and SY wrote the manuscript and conducted the researches relevant to the present paper. All authors read and approved the final manuscript.
